# Supplementary material for: Interprofessional Collaboration in Obstetric and Midwifery Care—Multigroup Comparison of Midwives’ and Physicians’ Perspective
Source: Healthcare (Basel). 2025 Jul 24;13(15):1798. doi: 10.3390/healthcare13151798 (PMC12346844; doi:10.3390/healthcare13151798)

Supplement - Multigroup assessment of interprofessional collaboration during midwifery & obstetric care

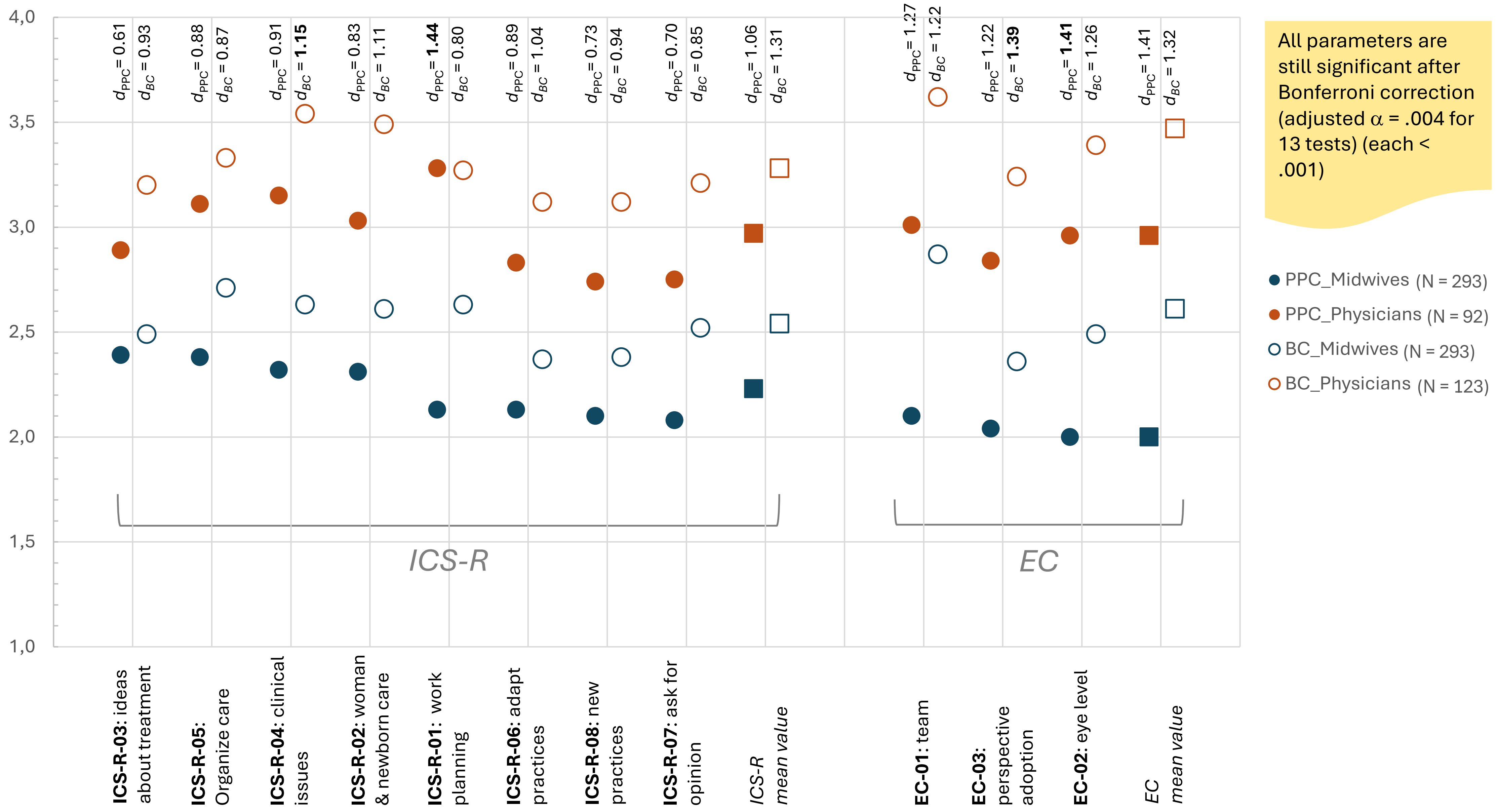

Supplement: Supplementary file 1 [file healthcare-13-01798-s001.zip › healthcare-3650727-supplementary.pdf]
